# Measuring Pleasure from Food—Validation of the Food Pleasure Scale by Multiple Techniques and Mixed Methods

**DOI:** 10.3390/foods13030477

**Published:** 2024-02-02

**Authors:** Nikoline Bach Hyldelund, Derek Victor Byrne, Wesley Dean, Claudia Squarzon, Barbara Vad Andersen

**Affiliations:** 1 Food Quality Perception and Society Team, iSense Lab, Department of Food Science, Faculty of Technical Sciences, Aarhus University, 8200 Aarhus, Denmark; derekv.byrne@food.au.dk (D.V.B.); barbarav.andersen@food.au.dk (B.V.A.); 2 Sino-Danish College (SDC), University of Chinese Academy of Sciences, Beijing 101408, China; csq@ifro.ku.dk; 3 Department of Food and Resource Economics, University of Copenhagen, 1958 Copenhagen, Denmark; wesleydean@ifro.ku.dk

**Keywords:** food pleasure, Food Pleasure Scale, construct validation, reliability, mixed methods

## Abstract

The development of scales and questionnaires to assess pleasure perception has gained prominence, particularly for evaluating anhedonia in mental disorders. The Food Pleasure Scale is a comprehensive tool exclusively dedicated to measuring pleasure perception from food and food-related experiences. This study aimed to evaluate the face validity and consistency reliability of the Food Pleasure Scale using a mixed methods approach. Twenty-two participants completed the Food Pleasure Scale questionnaire and participated in in-depth interviews to understand their interpretation of the scale items. The interview data underwent thematic analysis, and the quantitative survey data was compared to the qualitative interview responses. Results indicated a high level of understanding of all items in the Food Pleasure Scale, confirming its face validity and applicability. The mixed methods approach supported the consistency reliability, showing consistency between quantitative measures and participants’ explicit and implicit expressions of food pleasure. Furthermore, the study revealed a novel aspect related to food pleasure: the concept of “making an effort”. Overall, this study highlights the comprehensibility, validity, and potential of the Food Pleasure Scale in consumer studies. It effectively captures the subjective experience of pleasure derived from food and food-related encounters, making it a valuable tool for further research in this domain.

## 1. Introduction

It is widely believed that the majority of food intake is influenced by hedonic processes rather than homeostatic ones [1,2,3]. As a result, there has been a growing focus on studying the hedonic aspects of eating in food and consumer research. The common aim of these studies is to gain a deeper understanding of the complex decision-making processes related to food intake [2,4,5,6,7,8]. This research has provided a comprehensive yet fragmented understanding of the various factors that can impact subjective pleasurable eating experiences [6,9,10,11]. However, it remains unclear how individuals differ in the key drivers of their hedonic experience and to what extent food pleasure plays a role [9]. Therefore, it is crucial to adopt a holistic perspective on the drivers of food pleasure to fully comprehend the factors that influence consumers’ individual and flexible food choices and eating behaviors. Numerous scales and questionnaires have been developed to assess the perception of pleasure. These scales have primarily been used for clinical purposes to evaluate anhedonia as a symptom of specific mental disorders [12,13,14]. Four widely used and validated scales in this context are the Chapman Physical and Social Anhedonia Scale [15,16], the Fawcett–Clark Pleasure Scale [17], the Snaith–Hamilton Pleasure Scale [18,19,20], and the Temporal Experience of Pleasure [16,21]. These scales take a broad perspective on pleasure in life and do not specifically focus on pleasure from food. However, there have been recent developments in creating scales that evaluate specific aspects of the pleasurable eating experience. For example, the Health and Taste Attitudes Questionnaires assess consumers’ attitudes toward food’s health and hedonic aspects [22,23]. The Well-being Related to Food Questionnaire (Well-BFQ) measures well-being associated with food and eating habits [14,24]. Additionally, the Reward-based Eating Drive scale targets behavioral symptoms characterized by an excessive drive to eat [25,26]. While several scales touch upon the pleasure derived from food and food experiences, a comprehensive and validated scale solely focused on all aspects of food pleasure has yet to be developed and fully validated. Developing and validating an efficient and accurate scale for measuring food (an)hedonia will facilitate future research on what individuals find pleasurable in food-related contexts. It will also help identify inter-personal differences, individuals with impaired hedonic responses and the exploration of the link between drivers of pleasure, hedonia, and food choices. Lately, a conceptual framework for developing a comprehensive measurement tool called the Food Pleasure Scale (FPS) was presented [9]. This scale aims to capture qualitative and quantitative aspects of subjective food pleasure. By consolidating these aspects, the framework provides a deeper and more nuanced understanding of food pleasure. It suggests that food pleasure is composed of multiple dimensions, such as the sensory properties (Appearance, Odor, Taste, Texture), collative properties (Familiarity, Novelty, Food variation), post-ingestive sensations (Mental wellbeing, Physical wellbeing, Sensory satisfaction, Surprises), expectations and desires (Memories, Needs, Choices, Habits, Expectations), Product information (Ethical values, Origin, Product information, healthiness), and the eating context (social setting and physical setting), which contribute to the overall hedonic response before, during, and after food intake. The framework also hypothesizes that individuals may experience dimension-specific anhedonia related to food pleasure. Additionally, the scale proposed in the framework incorporates a behavioral approach to explore variations in food pleasure further.

The current authors used an early version of the FPS in multiple consumer studies [27,28,29,30]. These studies were all designed as consumer surveys, and each focused on clarifying the scale’s usage potential and sensitivity by clarifying regularly occurring differences among different consumer segments and if disease-related anhedonic traits were associated with food-related anhedonic traits. For example, one study focused on cross-cultural differences in drivers of food-related pleasure [27], and another examined consumer segments’ characteristics in relation to the main drivers of food pleasure [28]. The remaining two focused on presumed anhedonic consumer groups, specifically individuals experiencing chronic stress [29], and whether anhedonic traits associated with depression or anxiety could be recognized in drivers of food-related pleasure [30]. These studies have demonstrated the applicability and appropriateness of the FPS for various scientific purposes and contexts. However, it is crucial to evaluate the comprehensibility, content validity, and ease of use of the FPS by end users if it is to be continued and recommended for further use in consumer studies. The current study aimed to assess the face validity and consistency reliability of the FPS in measuring food pleasure preferences among a non-anhedonic adult consumer group. A mixed-methods approach was employed to achieve this, comparing quantitative FPS survey data with qualitative interview responses. In-depth interviews were particularly relevant for this study, as they allowed participants to express their experiences freely and extensively regarding using the FPS and their individual food pleasure preferences. This approach facilitated the collection of qualitative data to evaluate participant acceptance of the existing instrument’s content and format and to explore any new aspects of food pleasure perception that were deemed relevant to consumers. Therefore, the specific objectives of this research were to perform the following:

- Examine the initial face validity of the individual questions and items of the FPS through qualitative interviews.
- Investigate the consistency reliability of the FPS by comparing results from survey data gathered prior to individual interviews with insights collected during the interviews.

## 2. Materials and Methods

### 2.1. Study Design 

**Figure 1:**
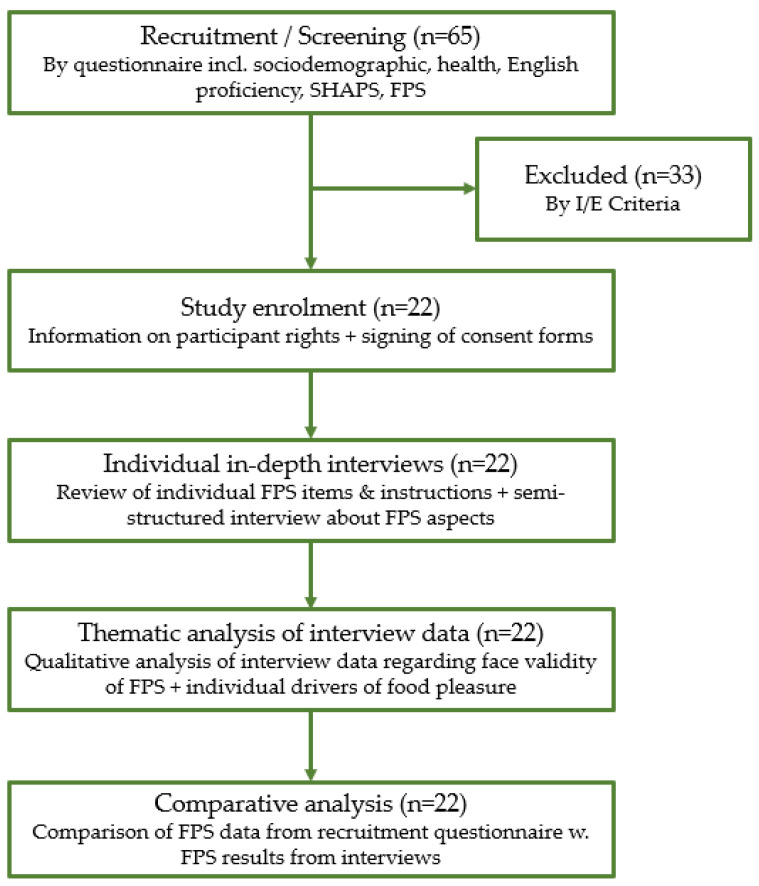
Flowchart of experimental procedure.

Participants in this study underwent two consecutive steps in a mixed-method design. Initially, they completed a short questionnaire, which included the Food Pleasure Scale, serving both as a recruitment tool and a data collection method. Subsequently, participants engaged in individual in-depth qualitative interviews, with an approximate one-month gap between the two activities. Both the survey and interview took place in the participants’ chosen at-home setting, ensuring comfort and a sense of security during the process. This setup facilitated video recording and the option to show participants pictures during interviews. The study received an exemption from ethical approval, as per the guidelines of the National Committee on Health Research Ethics in Denmark [31]. Written consent was obtained from all participants before commencing both the questionnaire and interview, accompanied by an oral explanation of their rights and information on GDPR regulations. Interviews were conducted via the online video conference tool Zoom, Zoom Video Communications Inc., Version 5.15.7, 20303 (San Jose, CA, USA) [32]. Moreover, this study followed the consolidated criteria for reporting qualitative studies as recommended by Tong and colleagues [33]. See Figure 1 for a complete and detailed overview of the study design.

### 2.2. Recruitment and Questionnaire

Participants were recruited via social media, with Facebook as the central platform, in August 2022. The questionnaire survey was administered online via the SurveyXact software, Version 13, Rambøll Management Consulting (Aarhus, Denmark) [34]. Information from the questionnaire was, in part, used to select participants for the in-depth interviews and to collect data. Thus, the questionnaire asked participants to indicate whether they had any dietary restrictions or diseases that might influence their dietary patterns or pleasure preferences. Furthermore, they provided demographic information (gender, nationality, age, educational level), English-speaking proficiency, self-reported diet type, and answered the Snaith–Hamilton Pleasure Scale (SHAPS) to assess general anhedonic tone. The SHAPS consists of 14 self-report statements rated on a 4-point ordinal Likert scale [18,19]. Any participants with a SHAPS score > 2 would not be considered for the study as such a result would indicate an abnormal general sense of pleasure [18]. Anyone above the age of 18, with no diet restrictions and an English-speaking proficiency corresponding to ‘upper intermediate’ level or more, would be considered for the interviews. The Food Pleasure Scale was included in the questionnaire for two reasons: (1) To allow for a strategic selection of interview participants, where a broad spectrum of food pleasure profiles was covered. (2) To make possible an evaluation of the consistency reliability of the scale. A complete transcript of the questions included in the Food Pleasure Scale can be seen in Table 1. In the questionnaire, the participants were asked to assess whether, in the moment of eating, they, in general, experience pleasure from each of the 21 items of the FPS (Question A). This section was followed by a rating of each FPS item in terms of how important that item was to their sense of pleasure when eating. They rated this by a 100 mm Visual Analog Scale (VAS) anchored by ‘Not important at all’ and ‘Extremely important’ at the extreme ends (Question B). 

**Figure 2:**
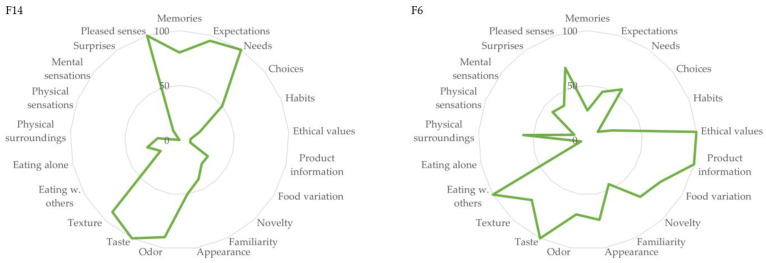
Food Pleasure Scale profiles of Participants F14 and F6. The radar charts show individual ratings of Food Pleasure Scale items on a 100 mm Visual Analog Scale.

Initially, sixty-five individuals expressed interest via the online survey in participating in the study; however, a final sample size of approx. 20–25 was pursued for the current study. This sample size was chosen based on previous studies using similar methods [35,36,37,38]. Thus, by means of purposive sampling, with a focus on selecting a broad range of participants across age, gender, different diet types, and FPS results, twenty-two final participants were selected. Special attention was given to their individual food pleasure profiles to make sure as many different nuances of the scale as possible would be covered in the interviews. Figure 2 shows the FPS profiles of two of the participants to visualize how the profiles varied and were selected, thereby ensuring a broad selection of FPS profiles were present among the study participants. A full overview of FPS profiles of all study participants can be seen in Table S1. In addition, a complete overview of FPS VAS ratings for each participant can be seen in the Results Section 3. Before commencing the interviews, written informed consent was collected from each participant. 

### 2.3. Pilot Test

A pilot test was performed to evaluate the flow of the interview guide and the individual questions. When conducting in-depth interviews, a pilot test is recommended to ensure the construct validity and reliability of the chosen data collection method [33,39,40]. Three people were recruited among colleagues for the pilot test. They had different ages, genders, and educational backgrounds; however, none had prior knowledge of the study or the use of the FPS. The interview guide was updated based on the input provided by the pilot test participants. Overall, no significant problems were identified. The feedback indicated that the initial format of the interview guide had room for improvement in terms of preventing any potential bias in the participants’ answers. As a result, it was determined that the interview should begin with an evaluation of face validity, followed by a section focusing on the factors driving the participant’s enjoyment of food. Further details about the interview guide can be found in Section 2.4.

### 2.4. Interviews

All interviews were conducted via an online video call by the first author, a Danish female Ph.D. student from the Department of Food Science at Aarhus University, Denmark. The interviewer had received guidance and supervision in terms of conducting semi-structured interviews prior to the interviews, and she had prior training and experience with conducting in-depth interviews. The interviewer had no prior knowledge of the background or personal beliefs of the participants nor any relationship to any of the participants. Most of the participants were interviewed alone. However, if permitted by the participant, a fellow researcher sat in on some of the interviews. This person made sure to stay as discrete as possible and was there to observe how the interviews went. All interviews were carried out during October 2022. The 22 interviews ranged from 1–2 h, with a total of 29 h and 45 min of video material. No repeat interviews were conducted, and transcripts and notes were not returned to participants.

The interviews followed a semi-structured guide comprising three parts. Firstly, there was a brief introduction section to ‘warm up’ the conversation, where the interviewer introduced herself, reminded the participants of their rights and asked the participants to share their relationship with food. Secondly, there was a detailed review of the Food Pleasure Scale (FPS), exploring the participant’s understanding of each item, clarity of instrument directions, and wording. Follow-up questions such as “Can you give me more details on that?” were used to gather additional information on understanding the items in the FPS. Thirdly, a section on the participant’s personal food pleasure drivers began, starting with an account of a recent pleasurable food experience. This question was included to get a first implicit impression of the participant’s food pleasure preferences as explained in his/her own words. Afterwards, six slides were presented individually, each containing pictures intended to depict the twenty-one items of the FPS. Each slide corresponded to one of the original dimensions proposed in [9], along with their respective items. This grouping approach was selected to maintain flexibility in the conversation, enabling participants to discuss multiple aspects if desired during the interviews. The slides can be seen in Table S2. When shown a slide, he/she was asked to explain what the pictures could mean to him/her in relation to enjoying food and meals. After reviewing all slides, he/she was asked to assess which item(s) were most and least important in terms of experiencing food pleasure. This allowed an explicit report of his/her food pleasure preferences to be attained. The interviews concluded with a short debriefing, offering participants a final opportunity for comments or opinions.

### 2.5. Data Analysis

The interviews were all video recorded via the utilized video conference software Zoom, Zoom Video Communications Inc., Version 5.15.7 (20303) (San Jose, CA, USA) [32]. Furthermore, notes from the interviewer were included as data material. A theoretical thematic analysis approach was utilized to analyze the interviews. This approach allowed for a flexible and comprehensive analysis across all participants, as outlined by Braun and Clarke [41]. Additionally, the items of the FPS served as a theoretical framework to guide the identification of themes. Moreover, the analysis encompassed themes such as the “most important aspects of food pleasure” and the “least important aspects of food pleasure”. Furthermore, a semantic and realist approach was adopted, assuming that the participants’ experiences and language genuinely reflected their reality [41].

Researcher triangulation was employed during data analysis, involving two independent researchers in the thematic analysis process to ensure comprehensive coverage of insights and minimize bias. This approach aimed for a more robust and objective analysis by avoiding reliance on a single researcher’s perspective [42]. Both researchers independently reviewed video material, extracting quotes and transcribing to identify emerging themes. The two separate analyses were compared in relation to observed key themes. Disagreements prompted a reevaluation of video material and discussions until a consensus was reached. A theme was deemed ‘key’ if it recurred frequently across the dataset and captured essential messages aligned with the research goals. 

Participants were asked how they understood each item to assess the face validity of the FPS. Overall coherence was evaluated by comparing individual responses with the intended meaning of each item. The number of participants whose responses aligned with the intended meaning for each FPS item was recorded, and key quotes were extracted to support the analysis. No quotations were extracted to support the reported secondary results to maintain clarity in the analysis. Moreover, for specifically analyzing the consistency reliability of the FPS, a comparison was made between quantitative data collected through the recruiting questionnaire and thematic analysis of the interview’s third part. This analysis involved comparing the quantitative ratings of each FPS item on a Visual Analog Scale (VAS) with insights obtained from the qualitative interviews. The comparison was made at an individual level, examining each participant’s ratings of the “importance of FP” for specific items and their corresponding explanations provided during the interviews regarding the most significant aspects of food-related pleasure. The items that fell within the 4th quartile of the quantitative data, representing the most important items for each participant, were compared to both explicit and implicit assessments of the most important FP aspects from the interviews. Similarly, the 1st quartile of the FPS VAS ratings, representing the least important aspects of FP, was compared to explicitly expressed responses from the interviews. Following this, an evaluation of congruity between the data was conducted. Quotes were pulled from the participants’ descriptions of a ‘special meal’ to provide clarity and exemplify the findings of the comparative analysis.

## 3. Results

### 3.1. Participants Demographics

A total of twenty-two participants were included in the study. Sixteen (73%) were women, and six (27%) were men. The average age of the participants was 28.14 ± 5.70 years, ranging between 21–49 years. All participants were residents of Denmark; however, their nationalities varied. Twelve participants were Danish (55%), whereas the rest came from a mix of different countries: two Poles (9%), one Hungarian (4%), one Greek (4%), one Portuguese (4%), one Italian (4%), one Lithuanian (4%), one Romanian (4%), one German (4%), and one Canadian (4%). Twelve participants had a long higher educational background (55%), whereas three had a medium higher education (14%), three had a short higher education (14%), three had a high school degree (14%), and one had a vocational education (4%). Regarding dietary type preferences, fourteen of the twenty-two characterized themselves as an omnivore (64%), five participants regarded their diet as flexitarian (23%), one as vegetarian (4%), one as vegan (4%), and one as carnivore (4%). A full overview of sociodemographic characteristics, as well as diet type for each participant, can be seen in Table 2. In addition, Table 3 shows the complete overview of each participant’s individual ratings of each Food Pleasure Scale (FPS) item. 

### 3.2. Face Validity of the Food Pleasure Scale Items

#### 3.2.1. Instructions

The FPS uses the phrasing: “In this moment, how important is the following to your experience of food-related pleasure?” The 21 FPS items were individually presented individually for participants to rate. To ensure clarity, participants were asked to assess the phrasing, and all found the overall question of the FPS clear and understandable.

M1: “I think they are pretty straightforward.”

F10: *“Well, I don’t think for me, it was difficult to understand. In my head, I think I understood them pretty well.”*

M3: *“Well, I think they’re [the questions, edit.] quite all right. There was just one thing towards in the beginning that it made me a bit more confused than the others. But the other ones, I think, are quite self-explanatory. And it’s quite easy to understand what they relate to.”*

F8: *“I think they are quite clear to me. Sometimes there is like so many factors in one that it is difficult to think of all things that are connected.”*

#### 3.2.2. Item Evaluation

Overall, the participants found most of the items of the FPS to be easy to comprehend and transparent. Variations in terms of interpretation did, however, occur, with a few items proving to be less clear than others. A more in-depth analysis of the face validity of each item is presented here. 

**Sensory properties:** The items relating to the sensory experience of eating, Appearance, Odor, Taste, and Texture were all completely clear to all participants. No one had any specific comments for these items, except that they were all self-explanatory and easy to relate to the perception of pleasure from food.

F7: *[About appearance] “Just how the food is presented.” [About odor] “And that’s the smell of the food.” [About Taste] “That’s what it tastes like.” [About texture] “Yeah, quite clear. If it’s crispy or smooth, or like that, right?”*

F8: *[About appearance] “So, it’s about how the food looks like.” [About odor] “That’s about the smell of the food. If it is like intense, or maybe it’s not so strong.” [About Taste] “So, that’s about flavor… Maybe it’s like a bit too salty or too sweet, or it’s lacking some flavors that I would expect in it.” [About texture] “So, it’s about the mouthfeel. So, when I chew the foods. That’s like the texture to me.”*

F9: *[About appearance] “So, how the food looks.” [About odor] “How it smells.” [About Taste] “How can I say it in another word… If when you actually eat, if it feels like sour, bitter or salty or whatever.” [About texture] “It’s like if it’s a liquid, or if it’s a solid or what shape it has.”*

M3: *[About appearance] “As a lot of chefs say, you first taste with your eyes. So, its presentation is important.”, [About odor] “It’s always nice to smell the food before you go in. It somehow enhances the taste of it.”, [About Taste] “Quite obvious, it’s important for you to like the food that you have made or that you are eating.”. [About texture] “You want it to be in accordance to what you like. For example, if you cook a piece of steak, and you want it to be, I don’t know, in a certain way, and it turns out tough. That’s not ideal.”*

**Expectations and desires:** The items Memories, Habits, Expectations, Choices, and Needs were all included in the FPS to reflect different aspects of having certain expectations and desires towards a meal or food experience [9]. In general, these items were understood as intended. 

Memories were intended to be understood as fond memories of particular food experiences that can bring pleasure to a similar meal or a meal that reminds them of that specific memory. Most of the participants (n = 19) understood it as such.

F15: *“I understood it as that I try to recreate food, which is going to give me the same pleasure as I’m connected to different foodstuff.”*

M5: *“I want to mimic some experience I had earlier with some food. That could be food I had when I travelled. That’s an example. I want to remake that food to remake that experience. Oh, and the classic one with childhood food. Like, we want to cook that like mom did or grandma did.”*

F2: *“I understand the question like… How important are the memories you like, which are linked together with some foods? And how important is it to you to remember certain memories, when you eat a certain kind of food.”*

F6: *“If there’s a certain smell that reminds you of a food related memory or a person or something like that.”*

The item Habits represents the pleasure you would get from maintaining a food-related habit. This is also how the participants understood it; however, one participant related habits to the concept of Familiarity (not quoted).

F11: *“I think that is important. Yes, I think there are specific things… like I do lemon water in the morning, for example. And so, I think that’s one habit that is like preparing me for the day, and it’s something that is so simple… Yeah, so I do value those things.”*

F9: *“Do you have like a daily or maybe weekly or monthly eating routine. So, specific type of foods that you would like to repeat and eat continuously.”*

F1: *“Food is very useful to have a routine with… To eat the same thing in the morning or to have this… Something that feels supportive of your day.”*

F12: *“Just eating the same thing over and over again. I don’t know… It’s like, you do your shopping routine, where you always buy the same things. Some people like that… To always eat the same meal all the time.”*

All participants interpreted the item Expectation as intended. Expectations should be understood as the pleasure you receive when your expectations towards a given food are met.

F16: *“For me, that relates to, when I see the meal, what I expect it to taste like and… the whole experience of it. If what I think beforehand, is also fulfilled when I actually eat the meal.”*

M1: *“For example, when you get a steak or something, and you are expecting it to give pleasure from… like the sensation of biting into the meat. A certain texture and certain meaty flavor.”*

M2: *“The way we value the food is also in accordance to our expectations regarding those products. So, we cannot value it individually without considering the individual expectations regarding it. Because that of course, will have a direct influence in the way we scale it or assess it.”*

F5: *“Yes, it’s like, what do you think the food will be like, and how does it fulfil the expectations or thoughts you have prior to eating.”*

The item Choices was meant to be understood as the aspect of having the ability to choose for yourself what you eat, which can bring pleasure to a meal. The majority understood this as intended (n = 20).

F3: *“Yeah, I think that the more variety you have, then the happier you are. Even when you are at a supermarket, I think the same…. When you are in a canteen and you see a lot of options, whether it’s vegan, vegetarian, meat, or fish. So, you have, I don’t know, freedom to choose.”*

F9: *“To have the opportunity to choose between maybe different dishes or different meals when you’re, for example, having dinner with your friends or by yourself. So, to choose by yourself which food you would like to eat.”*

F5: *“If I can choose to like… have something different. Is it a buffet? Can I add salt? Pepper?”*

F7: *“So, a good example of that would be my lunch situation at work, where it’s important that you can decide what you want to eat, and you know what’s in it, and you can make a choice between something healthy, unhealthy and all of that.”*

Having your needs fulfilled by food and the satisfaction that brings is proposed to be represented by the item Needs. Many different examples of types of needs were given in the interviews, thus proving that this item was also clearly understood as intended.

M6: *“Maybe I feel pleasure if the food that I eat fulfill my cravings at some time. Maybe not because I’m craving something sweet or fat…, but more like… also maybe based on my emotions. By my emotional state.”*

F1: *“That kind of explains itself, I guess. It’s nice to have the right to choose and then pick according to your needs. Otherwise, the needs could not be fulfilled besides the hunger. Maybe it’s about the nutritional value or specific tastes.”*

M1: *“For example, if you’re hungover, and you want a slice of pizza. It could be in that matter, like comfort food. Like when your body feels like it needs something fatty or spicy, or if you feel you need to get some salt into your body.*

M2: *“Eating in itself is like a basic need. So, of course, considering that dimension of food is also important, because we don’t eat only to derive pleasure from it. We also have some needs, we have… like we need energy or we need to feel better with ourselves. That’s a need in itself.”*

**Collative properties:** Familiarity, Novelty, and Food variation are all aspects that reflect the collative properties of a meal [9]. 

Familiarity should be understood as the aspect of a food or meal that brings pleasure by being familiar, recognizable, and known to them. This seemed clear for most participants (n = 19), although some participants (n = 3) explained how that item was also related to having good memories about a meal (not quoted).

F10: *“So familiar foods… that would be for me… I think about the foods that I grew up with. The foods that I have known for my whole life, from childhood. Lasagna, for example. Yeah, that that’s what I think of.”*

F3: *“Something familiar that kind of takes you back to your home country. Or a memory, or whatever, of what your mom made for you.”*

M2: *“That sense of familiarity with the food makes it much more special… It really goes back to, remembering people that you love, probably that they are not present anymore, or you’re distant from a place that you really value.”*

F13: *“Like experiencing those dishes that we know really well, and that somehow gives us a memory of good times… And that we re-experience something that we already know. Maybe it’s something from our national traditional food cooking.”*

Novelty should be understood as the aspect of a food or meal that brings pleasure by bringing new or unknown impressions. This was likewise clear to most of the participants (n = 20), yet some participants (n = 2) expressed that they found it hard to distinguish the item of Novelty from the item of Food variation (not quoted).

F5: *“Is it something, I haven’t tried before, and especially tastes I haven’t tried before.”*

F8: *“I think it’s quite simple. So, if I haven’t tried something before, then I would say it’s new. Or maybe I know the ingredient, but the way of preparing the dish out of it, it’s new to me.”*

M6: *“That’s to expand my horizons food wise. So, for instance, trying different dishes from across the world instead of just eating the same old bland Danish food.”*

M3: *“I think, yeah, this is quite self-explanatory. So, your openness, and how important it is to you to try new things. So, you introduce some spice into your life in general.”*

Lastly, the item Food variation, which entails the pleasure one might get from experiencing a variation of different foods, also proved to be understood as intended. However, some participants found it slightly confusing and overlapping with other items, such as Novelty (n = 3) or Choices (n = 3) (not quoted).

F13: *“Yeah, I come to think about differences in tastes and in textures and in smell and so on. And also, being a bit challenged on how we usually eat or…”*

F15: *“That you take in consideration the different groups of foods. So, you make sure that there’s always some fiber, protein, carbs, fats. I think that’s how I get it.”*

F5: *“It could be according to the taste, but also according to what it contains.”*

M3: *“You don’t want to eat the same thing over and over. So how important that is to you, to not eat the same thing every day because…it kills your spirits.”*

**Eating context:** The items relating to the context within enjoying a meal; Eating w. others, Eating alone, and Physical setting all seemed entirely transparent to the participants. Especially, the items reflecting the social aspects of a meal were fully understood as intended. Thus, correct examples of the pleasure one might experience from Eating with others and Eating alone were given.

F3: *“I think it’s very nice [to eat with others, edit.]. It makes you enjoy your food more. You kind of enjoy the food together, so you’re less selfish.”*

M2: *“Food, of course, has a social dimension. For instance, like eating with the family, eating with friends… Food is what blends in with everyone, [what makes people, edit.] get together to socialize, and food is the intermediary.”*

F13: *“To me, the pleasure of eating with others is about the conversation you are having. Sharing the dining experience and the impressions of the food.”*

F14: *“So, food and how I perceive it and the pleasures I get from it when I’m with others… And I weigh that against when I’m eating by myself, for example.”*

F11: *“I think it’s something that I have learned [to eat alone, edit.] as I get older. That it really can be very satisfying to even sit alone in a restaurant and enjoy a good plate of food. And it’s something that I think more people should do, and it’s something that is a little bit daunting and scary. But there is a lot of pleasure in actually enjoying the food by yourself, because then it’s not everything else that’s happening around you… Like I go through stretches where I’m just having like a salad or easy things and then taking the time to prepare a meal for myself… I feel like it’s a self-care moment. And there’s pleasure in that for me as well.”*

M4: *“Yeah, I mean, it’s interesting because it can be very different. Sometimes I enjoy a lot to also eat alone because it makes me more aware. Also, just to enjoy myself and enjoy my food. But of course, it’s very, very, very nice to eat together with people and sit around a table.”*

The item of Physical setting was also clearly interpreted by all participants and was understood as the pleasure you get from the physical surroundings when eating. Many examples of different physical settings were mentioned, proving the item was transparent and easily recognized.

M5: *“That’s the environment you’re eating in. At home…Or at a restaurant.”*

M6: *“That I feel pleasure when I eat food in a specific context. So, the physical surroundings could be, let’s say, at home, at work, in the nature, by the sea, and the forest and so on. That’s how I understand it.”*

F3: *“I feel like when I eat sometimes on the couch or in the kitchen, then I’m not really influenced by the environment, and sometimes I think it makes me eat faster. But if I am at a restaurant or… I’m the park, which is a nice place, then I feel like I want to enjoy my eating, and just like…look at the surroundings and just be in the moment.”*

F4: *“I mean, clearly, there’s a difference between eating a meal at a train station, and somewhere where there is some nice service. But also, it’s really nice just to have breakfast in bed.”*

**Product information:** Participants found the items related to the pleasure of knowledge about food product characteristics—Product information and Ethical values—completely clear and easily comprehensible. Various examples provided by participants indicated the clarity and adaptability of these items to individual experiences.

F1: *“It’s very important to know where the food comes from actually, and to have a lot of information available.”*

F2: *“I think that’s about if you value, for example, [knowing, edit.] how many calories is in the food and how much sugar, fat, or protein.”*

F7: *“That would be if it’s organic or not. Or maybe some religious restrictions if someone has them. Or if they’re vegetarian and they don’t want to eat meat for different reasons. Or if the food is sustainable or comes from places where nobody was hurt while getting the food or producing it.”*

M4: *“[It is, edit.] really good that you can use these web pages now with help line, which has a lot of information about the different kinds of foods. So, I enjoy it a lot to inform myself about the things that I consume.”*

Likewise, Ethical values appeared to be easily related to pleasure from food.

F11: *“I think it’s really nice, when you can connect to your food through kind of the ethics, community as well. And I worked at farmers markets, for example, and so kind of knowing the practices behind how food is grown or produced, I think that just adds to pleasure. And I think it’s kind of this mix of passion and pleasure and kind of having access to that is really… pleasing.”*

M2: *“So, even if I eat meat, I just want to make sure or at least have the mental comfort of knowing that this animal wasn’t mistreated or something. But of course, it can be much more than that… I’m just providing an example of the ethical standards, that I think of.”*

M6: *“I guess it’s all about that I feel better when I eat something that doesn’t compromise different types of ethics.”*

F14: *“Yeah, whether or not I will consider it, when I’m buying things or eating. That’s how I interpret the question… Like if I were to be ethical, especially towards like animal welfare or cruelty, and like organic foods. And sustainability is also, I think, linked to ethics in some way.”*

**Post-ingestive sensations:** The items of Physical sensations, Mental sensations, Pleased senses, and Surprises reflect different retrospective sensations of pleasure from eating food [9]. The participants gave many different examples to describe Physical sensations, thereby underpinning that this item was fully comprehended as intended. A few participants (n = 3) had trouble distinguishing Physical sensations from Mental sensations, though (not quoted).

F8: *“So, that will be about like the temperature of the food. So, in winter, I really like to eat soups and really warm things. So, the temperature. And satiety, of course, as well. Maybe after very sugary or salty food, I will feel very thirsty. And maybe if I have too much sugar, I feel this sugar rush.”*

F2: *“If you think it’s important that you have a nice feeling in your body afterwards. For example, if you’re feeling full or not.”*

F4: *[Talking about eating cake, after a long period of abstaining from sugary foods, edit.] …“So that was just really the ultimate when you’ve been without [a certain food, edit.], and then you get something that gives you that kick again, you know. Yeah, you have that pleasure then.”*

F12: *“I start to think about the food that makes you… like some food makes you almost drunk, and maybe because there is alcohol in, but also other foods have like a strong aphrodisiac. So, food that somehow is uplifting.”*

Likewise, Mental sensations were understood by the majority as the different sensations or feelings of well-being one might have after eating. Thus, this item also seemed transparent to the participants. A few participants (n = 3) expressed that they were unsure about the meaning of this item (not quoted).

F2: *“I think that’s if you are feeling more happy or more relaxed after eating, and if you value that highly or not.”*

M1: *“For me, when I eat carrots, or something really green like a green kale, I feel that my mind is… I don’t know… it’s ‘quicker’.”*

F4: *“I like helping my husband make a successful meal, and then… Or he has made a successful meal, and we eat it and it’s like…Mmm [enjoyable sound, Edit.]… It’s just… It’s amazing. But then again, when he does that, I would so much love to share it with everybody because of what I feel.”*

F12: *“I think that as the food that somehow brings you into a certain mood after eating it.”*

The item Pleased senses was intended to be understood as the collected pleasurable sensation of having all senses stimulated in a pleasing way. Nineteen participants fully understood the item Pleased senses as intended, whereas the remaining three expressed confusion about the meaning of it (not quoted).

F9: *“So, when you’re eating, if you actually are paying attention to all your senses, and if you like to satisfy all your different senses, like smell and taste and the view and everything.”*

F6: *“If you get all your senses involved when you have a meal, then I think that’s what makes a better meal.”*

F7: *“That would be when we when we eat something, maybe with our fingers, and it gives a different sensation than eating it with a fork. And yeah, it’s like combining all the senses to experience it.”*

M6: *“I guess it’s... All of the different things about the food, I’m eating, altogether that satisfy my needs or fulfill my expectations. Yeah, and then that is resulting in me feeling some pleasure.”*

Finally, the item Surprises, which represented the pleasure one might get from being surprised by a food or meal, was generally interpreted as intended by all participants.

M6: *“[When, edit.] you eat a certain type of food, and you have some specific expectations, and then you actually experience something that you didn’t expect… And that makes you feel pleasure.”*

M1: *“If all of a sudden there’s something sweet in what you expect to be salty. Or if there’s some crunch inside the soup. Or if it looks like it’s supposed to taste or I feel like something, and then it isn’t.”*

F16: *“That would also relate to the expectations I have for the meal. And if it’s then a positive surprise.”*

F12: *“That one would be a bit like eating something that looks like an orange and then it tastes like an apple.”*

In summary, the study participants reported a high level of understanding of all items in the FPS. Furthermore, they provided specific and detailed examples. This suggests that the study participants could effectively use and engage with the scale as intended, thereby verifying the scale’s high degree of face validity.

### 3.3. Consistency Reliability Evaluation of the Food Pleasure Scale

A comparative analysis was conducted between the results of the quantitative VAS scale ratings of each FPS item (see Table 3) and insights from the qualitative interviews to evaluate the consistency reliability of the FPS. This analysis was performed at an individual level, comparing each participant’s item ratings regarding the “importance of FP” to the explanations they provided during their respective interviews regarding the most significant aspects of food-related pleasure. Items rated in the 4th quartile, representing the most important for each participant, were compared to both explicit and implicit assessments from interviews. Similarly, the 1st quartile of FPS ratings, representing the least important aspects of FP, was compared to explicitly expressed responses from interviews. An evaluation of congruity between the data was then performed. 

#### 3.3.1. Most Important Food Pleasure Scale Items: Congruence of Interview and Questionnaire Responses

Overall, the consistency reliability was determined to be high. Specifically, 86% of the participants (19 out of 22) consistently indicated a high level of importance in the same aspects of food pleasure that they had initially rated highest in the questionnaire. This finding suggests a strong consistency in their responses over time. Furthermore, when comparing the FPS questionnaire results to the implicit insights related to each participant’s recall of a ‘special meal’, again, a high consistency reliability was detected. Here, 21 out of 22 participants (95%) gave accounts congruent with their FPS item ratings. Table 4 gives an overview of the results of this analysis. 

To illustrate the analysis results, selected quotes about a ‘special meal’ from participants are used, demonstrating the consistency and consistency reliability of the FPS through implicit insights. Participant F11, who had rated elements like ‘Memories’, ‘Eating w. others’, ‘Pleased senses’, ‘Appearance’, ‘Eating alone’, ‘Familiarity’, and ‘Novelty’ highly shared an experience of a social dinner with friends from various cultures. The eclectic mix of dishes, delightful food, appreciative atmosphere, and social company combined to create a beautiful and special meal, aligning with her high FPS ratings for ‘Eating w. others’, ‘Pleased senses’, ‘Familiarity’, and sensory aspects in general.

F11: *“Thanksgiving in Canada is coming up in the next couple of weeks, and so I have a friend here and his partner is Canadian, and so they celebrate Thanksgiving every year here (in Denmark, edit.]. I was invited for dinner at their summer house, and it was a bit of like a potluck style. So, we coordinated a plan, and I made two desserts… It was really interesting, because I think… Like the turkey is very much a North American like staple for Thanksgiving and having kind of like the fresh Danish potatoes and the brown gravy and then a baguette, and also potato chips to dip in the gravy at the end…. So, everyone brought a different kind of touch of their culture. And I thought that was a really beautiful way to kind of celebrate because the whole purpose of our ‘Friendsgiving’ dinner was really to kind of appreciate who was there, and what’s happened in the past year. And so, it’s very cozy that people kind of infused that with bits of themselves through their food.”*

Participant M4, who prioritized ‘Surprises’, ‘Appearance’, ‘Physical sensations’, ‘Habits’, and ‘Ethical values’ in the questionnaire, shared a pleasurable meal experience from a recent train ride. The interview revealed connections between his explicit and implicit food pleasure preferences, with emphasis on ‘Physical sensations’, ‘Surprise’, and sensory aspects during the train meal.

M4: *“So, I was fasting the last weekend… And then Friday, Saturday, Sunday, I was only drinking these juices. And then on Monday, I was taking the train, and I was breaking the fast with some blueberries, I had bought. I remember very clearly, I was eating the blueberries, so it’s not a special dish, I had prepared. But it was very nice to eat the blueberries, and also some apples a little bit later. It was a very interesting experience to…, because I’ve never fasted before in my life, so just to experience non-fluid food. It was very nice. [When asked to pinpoint the aspects, which gave pleasure, edit.] It was definitely the way that I could use my ‘chewing’. I mean, I was chewing the blueberries. And it was definitely not under the best surroundings, because I was in a train. But I still enjoyed it a lot, and I was just eating one blueberry by the other and very slowly.”*

Participant F13 rated ‘Food variation’, ‘Taste’, ‘Eating w. others’, ‘Novelty’, and ‘Expectations’ as the highest in the questionnaire. She described a celebratory atmosphere where pleasant company, meal context, food quality, and the restaurant’s decoration and environment blended harmoniously.

F13: *“I was at a board meeting at my workplace. In the break we went to a place just next to it called, where it’s actually just a wine bar, but they also serve food. So, we all sat in the kitchen, like in the restaurant kitchen at two long tables, and then he was like serving us each a dish of pasta with fennel. So, the fennel was cooked into the pasta sauce and also fresh fennel on top, and then different various wild mushrooms. Then we had a good cold beer to it. And the aesthetics of the kitchen that we were sitting in, was just like quite a beautiful little hut with wood on the ceilings everywhere. And it was very warm in there, with steam from the cooking. It felt very ‘at home’ being in his space, but yet it was also somewhere a bit extraordinary from a regular home. So that was a really good experience. We had some really good talks, and everyone was laughing, and it was a really good break from having very, very serious talks at the meeting. And then so there was this mood of kind of celebration on a regular Wednesday.”*

Participant F3 enjoyed lunch at the university library canteen, highlighting the unique pleasure derived from the presentation and ambiance. She particularly appreciated the variety of dishes, especially vegetable options. This aligns with her top-rated FPS items, including ‘Taste’, ‘Texture’, ‘Memories’, ‘Pleased senses’, and ‘Eating w. others’. Notably, ‘Appearance’, ‘Odor’, and ‘Novelty’ also closely matched her preferences, reinforcing the coherence between her implicit food pleasure preferences and qualitative FPS ratings.

F3: *“So yesterday, I was at the canteen of the Royal Library, and I think it was very good because they had a lot of variety, a lot of veggies made in different ways and so on. And it was very, very like broad in what you could eat. So, there were options for vegetarians or vegans, and so on. And then I had… Well, I kind of had a ‘high’ from those meals, because the veggies looked very, very good, like extremely good! … It was pleasurable that I had so much choice to choose from. And just like also how it looked. They made this decoration with the squash and so on that it was more for a fall season, I guess. So, it made you eager to try it. So that’s where I got the pleasure from. Like; ‘Ahh it looks so cute, I want to try it.’.”*

#### 3.3.2. Least Important Food Pleasure Scale Items: Congruence of Interview and Questionnaire Responses

An evaluation of congruity between the least important FPS items as rated in the questionnaire and what was explicitly expressed in the interviews gave a somewhat more unclear result. Only 11 out of 22 participants (50%) matched their questionnaire results with their interview testimonials. Table 5 gives an overview of the results of the least important food pleasure aspects. As the participants talked about positive experiences of food pleasure, it was not fruitful to implicitly assess which aspects were least important to them. Thus, no quotes will be presented here concerning this matter.

#### 3.3.3. Emerging Aspects of Food Pleasure

Several interview participants spontaneously highlighted an aspect of food pleasure not covered in the FPS: the effort put into meal preparation. Eight out of twenty-two participants mentioned that investing effort in a meal could enhance the hedonic eating experience and promote mindful eating. Participants M6, F6, F2, and F3 said the following:

M6: *[Talking about a special Christmas meal experience, edit.] “So, I think it’s just being together with your family and your nearest in your life, and enjoying the situation, looking forward to having a nice evening. You have to put in an effort to try to make a lot of different dishes or types of foods. And this is typical for only times like Christmas eve, because, you know, you spent a lot of time in preparing these foods.”*

F6: *[Talking about a picture of a modern salmon dish, edit.] “The one with the salmon looks super nice. Even though there’s a lot of stuff that I can’t really see what is. But I think that’s kind of fun. So, I wouldn’t mind eating something I didn’t know, what was. And you can see on the plate that there has been a lot of thoughts about how to prepare this meal. The salmon dish seemed like a lot of effort was put into presenting the meal in a visual nice way.”*

F2: *[Talking about a special meal she had] “I always love when, like the food has been prepared for a while, or like somebody really thought about what they wanted to make and really put some effort into it. I think that’s also because, then it becomes like a ‘thing’.”*

F3: *[Talking about a picture of a beautifully set dinner table, edit] “It’s probably for a dinner that still hasn’t happened. I like the way they decorated it…, and then just like seeing all the dishes laid out carefully. They made an effort to decorate, and then it would kind of make me more relaxed and it will make me eat less. That’s what I feel like when you see that they have made an effort and, you know, psychologically more or less, they have to sit down and enjoy and eat slowly.”*

Other participants used the aspect of *making an effort* to describe what happens to the pleasurable experience of a meal when no effort was put into it. For instance, participants F6, F16, and M4 said the following:

F6: *[Talking about a picture of a pasta dish, edit.] “The one with the pasta, with the ketchup on, and they tried to decorate it with some basil on top… It’s just…That’s just not that much effort into that one.”*

F16: *[Talking about a picture of a pasta dish, edit.] “I see the spaghetti with the ketchup... I feel a bit defensive in a way… I know that if people served the pasta for me, I would eat it, but I wouldn’t be happy. I would rather have something else. Yeah, even though they have the herbs on top. I just feel, it’s very lazy, in a way. That they… if people served this for me, I would feel that they didn’t put any effort into it, which would reflect on the whole experience; that they didn’t really want to be there and they didn’t want to cook the meal.”*

M4: *[Talking about a picture of a simple pasta dish and a modern salmon dish, edit.] “I mean, the pasta dish is really not something that is... There’s no complexity to it. It’s just cooked spaghetti with some sweet ketchup on it, and there’s really nothing in it also. So, this is mainly just sugar. So, I mean, this is the carbohydrates and then the ketchup, probably with a lot of sugar. So, in the end, it’s just giving you a very high glucose spike. But this is another important thing, because that would not be something I would think about with the other dish [a complex salmon dish, edit.], because then I would not think about blood sugar and everything about that. Because it just really looks like a composition and someone that has really put a lot of effort into a dish.”*

The mixed-methods approach used in this evaluation of the consistency reliability of the FPS provided valuable insights regarding the scale’s applicability and reliability. Overall, the study participants demonstrated strong consistency in their quantitative FPS measures and explicit and implicit expressions of key aspects of food pleasure. These findings suggest that the FPS exhibits good consistency reliability over time and across different research methods.

## 4. Discussion

### 4.1. Insights Regarding Face Validity 

One of the main aims of this mixed-methods study was to explore how individuals understand and interpret each of the 21 items of the FPS, as well as whether those interpretations were aligned with the original conceptual intention, as described by Andersen and colleagues [9]. If the items were not understood as intended, it would compromise the face validity of the FPS. The participants gave both precise and detailed examples illustrating their understanding of the FPS items. Moreover, most participants indicated that they found the scale instructions and items to be clear and self-explanatory; however, some items were less consistent with the original intention or overlapping with other items. For instance, Memories, Habits, and Familiarity overlapped for some, all relating to the comfort of familiar foods. However, most participants found it easy to distinguish between the three. Similarly, Novelty, Choices, and Food Variation overlapped for some. Here, some participants interpreted these items as representing aspects of food pleasure linked to having different and varying experiences of food, in some instances, as opposed to enjoying well-known or ‘basic’ foods. Pleased senses were generally clear, though a few participants had difficulty defining it. Physical sensations and Mental sensations were challenging to distinguish for some, possibly due to mixed interpretations. In general, these inconsistencies may arise from participants not being accustomed to reflecting on the pleasure derived from food, and some aspects may be a novel concept for them. This suggests that participants needed to delve deeper into personal interpretations during the interview beyond their initial responses in the questionnaire. In addition, it is commonly known that when answering a questionnaire, participants tend to grow weary depending on the length of the questionnaire and the number of repetitive questions within the survey. The questionnaire results are, therefore, more susceptible to response bias [43,44]. To avoid overlaps in the interpretation of individual items, some examples for these items in connection with prompting the questions could support their correct interpretation. The authors have previously used this approach [27,28]. However, if doing so, one must also be aware of the possible bias of over-directing the thoughts of the individual participant, thus possibly leading to answers that do not reflect the truly pleasurable aspects as perceived by the individual. Accordingly, the authors believe the items should be presented without examples because the overlap in the understanding of items was experienced by only a few participants, and most subjects clearly understood and separated the items.

### 4.2. Insights Regarding Consistency Reliability

The second aim of the current study was to investigate the consistency reliability of the FPS. This was done by qualitatively comparing the individual ratings of the importance of the FPS items from the questionnaire to assessments of explicitly and implicitly expressed food pleasure preferences. Insights on the consistency of the results obtained from the FPS were found. As shown in Table 4, 86% of the participants demonstrated good consistency between quantitative FPS data and what they explicitly expressed as the most important aspects of food pleasure to them in the interviews. Moreover, 95% of the participants showed good consistency between quantitative FPS results and their implicitly expressed food pleasure preferences, as assessed by their individual recollections of a ‘special’ pleasurable meal. Thus, the study proved good consistency reliability of the FPS. 

Inconsistencies emerged between questionnaire data and explicitly expressed interview responses regarding the least important aspects of food pleasure. These differences may stem from the different response types used—the questionnaire employed a 100 mm Visual Analog Scale (VAS), while interviews involved direct verbal opinions. The interview setting, allowing time for thought and potential influence from other topics, might also impact responses. Nonetheless, face-to-face interviews generally expect honest answers [40,45,46]. On the other hand, when answering a questionnaire, individuals rely on their interpretation of the questions and are guided only by the instructions provided. It is also important to note that self-report bias is commonly present in survey studies [44]. The minor inconsistencies may also indicate variations in participants’ own level of consistency in their food pleasure preferences. Certain items may be influenced more by contextual factors, like the physical eating environment, while others may be tied to personality traits, such as enjoying social company while eating. Therefore, it is possible that individual food pleasure profiles are dynamic and can vary in consistency across contexts and over time. Prior research on the relation between different mental disorders and (an)hedonia has suggested that anhedonic symptoms are either temporal or determined by trait-like characteristics, dependent on specific mental health states [12,47]. The original conceptual framework of the FPS considered the differentiation between trait and state (an)hedonia [9]. Thus, the FPS was designed with the understanding that these two aspects are interconnected and should not be disregarded. Consequently, variations in the perceived importance of different food pleasure aspects may naturally occur, reflecting trait-like or state-like hedonic tones at different life stages [9]. However, it is essential for the FPS to accurately distinguish between these two aspects. Despite these factors, consistent responses were observed in relation to the most important aspects of food pleasure for most participants, supporting the chosen mixed-methods approach and a high consistency reliability. To further assess the consistency reliability, conducting a repeated study using only quantitative data and a larger sample size is recommended, thus enabling statistical calculations of correlations and reliability. Nevertheless, the mixed-methods approach used in this study has demonstrated the scale’s reliability over time and its applicability as a scientific method in both quantitative consumer studies and qualitative interviews. 

### 4.3. Novel Insights for the Conceptual Framework of the Food Pleasure Scale 

A novel aspect of food pleasure, “Making an effort”, which initially was not included in the FPS, appeared in the interviews. Eight of the participants spontaneously brought this topic up as something that would enhance the pleasurable experience of a meal. To ‘make an effort’ when preparing a meal could be related to the anticipatory expectations that can be formed towards eating a specific meal. Thus, one could argue that ‘making an effort’ is simply a different version of ‘affective expectations’, where the degree of expected enjoyment towards a stimulus or food is equally as important as the actual pleasurable experience of consuming that item [48]. Some supporting quotes regarding this aspect of ‘Making an effort’ also bear witness to this overlap with the aspect of Expectations. Thus, it is recommended to consider whether future studies using the FPS should include this additional aspect or if the already included item, Expectations, simply encompasses it too.

Another interesting result, which was apparent from the individual recollections of ‘special’ meals, was that 18 out of 22 participants ascribed the social eating context to be of significance in terms of the pleasurable experience. This is interesting, as just 10 participants had Eating w. others among their top-rated FPS items. Perhaps an explanation for this could be that when asked to think of a special pleasurable meal, many people will think of situations where something extraordinary was present, such as different company than you experience on an everyday basis. The social context of a meal has, in previous studies, been found to significantly increase the satisfaction of a meal [49,50,51,52]. Likewise, one or more sensory aspects of food pleasure were apparent throughout the results regarding the most important aspects of the questionnaire and the interviews. This was somewhat expected, as other studies using the FPS have consistently shown that the sensory aspects of a meal are the primary driver of food pleasure [27,28,29]. Moreover, the sensory aspects of a meal have previously been shown to increase both the acceptability, liking, and satisfaction of foods [52,53,54].

### 4.4. Strengths, Limitations, and Future Directions

The current study used a mixed-methods approach to examine the content validity of the Food Pleasure Scale. This approach allowed for a comprehensive evaluation of participants’ understanding of the scale’s content and format and an exploration of new aspects of food pleasure that are relevant to consumers. The assessment of face validity using in-depth interviews was particularly valuable, as it provided a deep understanding of participants’ subjective experiences while completing the scale. This approach also allowed for an examination of how individuals interpreted the scale items and whether any items were confusing or redundant. These insights could be used to improve the scale and better capture the lived experience of the measured construct. Furthermore, the inclusion of a time gap between the collection of quantitative and qualitative data enhanced the assessment of consistency reliability. It is recommended to have a time interval of at least two weeks between testing to mitigate the influence of potential confounding variables, such as learning effects, which could potentially impact the data [55,56]. 

In presenting the findings of our mixed-methods study, it is important to acknowledge a limitation. The outcomes of this study were heavily reliant on the responses provided by the 22 participants involved. Consequently, the generalizability of the results to the broader population may be limited. Additionally, it is crucial to note that the findings solely rely on subjective reports and lack support from objective measures. The distribution of the study sample may be considered imbalanced when compared to sociodemographic variables and dietary patterns of the general Danish population. However, it is important to note that the selection of participants was primarily based on securing a wide range of FPS profiles within the study sample. Both sociodemographic and lifestyle factors may be skewed as a result of this. I.e., it is possible that certain FPS profiles are more likely to be associated with specific diet types, leading to an imbalance in the dietary groups. However, because the aim of the current study was not to obtain generalizable results, this was not considered to be a critical aspect of the study design or findings. One could hypothesize that the variations in sociodemographic factors, particularly the nationalities of the participants, may introduce bias in the study findings. However, these diverse variables were considered less pertinent for evaluating the face validity or consistency reliability of the FPS. Nonetheless, during the selection of study participants, it was acknowledged that the inclusion of a diverse range of cultures could potentially enhance the applicability and comprehensibility of the FPS. 

To further document scale reliability, it is advisable to carry out a subsequent study that focuses solely on quantitative data and incorporates a larger sample size (preferably representative) and run a test-retest study. This approach would allow statistical calculations to be performed, enabling the examination of statistical correlations and reliability. Yet, as the current study had an explorative approach and employed mixed methods, our sample was not intended to be representative. Nevertheless, incorporating the qualitative approach offered additional benefits, including gathering comprehensive insights into the user experience of the scale.

The combined results of the current study and former studies using an early version of the FPS [27,28,29,30] have proven the applicability of the scale for use in the assessment of both individual food pleasure profiles as well as profiling of consumer segments in different cultures and consumer groups. If the FPS is intended to be used as a diagnostic tool for individuals with anhedonic symptoms, it would be recommended to modify the questionnaire to allow for the calculation of an FPS score. This score could then be used to establish specific cut-off values related to anhedonia. On the other hand, the current construction of the FPS provides valuable insights into multifaceted consumer profiles, like what is observed in traditional sensory profiling. 

## 5. Conclusions

In conclusion, the findings of this study indicate that participants understood the individual items and questions of the Food Pleasure Scale (FPS) as intended and in line with the original conceptual framework. The participants also found the scale instructions and items to be clear and self-explanatory, although there were a few instances where certain items deviated from the original intention or overlapped with others. Additionally, the evaluation of consistency reliability showed good agreement, as the majority of participants demonstrated consistency in their expressed food pleasure preferences when comparing quantitative FPS measures to interview responses. This suggests that study participants can effectively use and engage with the scale as intended, making it applicable in consumer studies. Furthermore, a novel aspect related to pleasure from food, namely the concept of ‘making an effort’, emerged from the qualitative interviews. It is worth considering whether this aspect should be considered as an addition to the FPS or if it is already encompassed by the existing item ‘Expectations’. This consideration should be explored in future studies. In totality, the current findings offer evidence supporting the validity of the Food Pleasure Scale as a tool for assessing the subjective experience of pleasure derived from food and food-related experiences.

## Figures and Tables

**Table 1:** Overview of the questions, item-related statements, and response types included in the Food Pleasure Scale.

	Question A	Question B
Question	“Do you experience pleasure from the following aspects related to food and meals?”	“How important is the following for your experience of food pleasure when eating?”
Response type	Binary scale: “Yes”/“No”	100 mm Visual Analog Scale anchored by 0 = “Not important at all” and 100 = “Extremely important”
Item-related statements		
Memories	“When you have memories about food?”	“To experience positive memories of food”
Expectations	“When your expectations of food are confirmed?”	“To experience my expectations towards the food fulfilled”
Needs	“When your food-related needs are satisfied?”	“To experience satisfying a need”
Choices	“From having different foods to choose from during a meal?”	“To experience having choices in the eating situation”
Habits	“When you can maintain a food-related habit?”	“To be able to maintain a habit”
Physical setting	“From the physical surroundings around the meal?”	“To experience positive physical surroundings”
Ethical values	“When your ethical values to food are met?”	“To fulfill ethical values about food”
Product information	“When you can obtain product information about the food?”	“To experience having desired product information about the food”
Food variation	“When you experience food variation?”	“To experience food variation”
Familiarity	“When you experience familiar food?”	“To experience familiar food”
Novelty	“When you experience new/unknown food?”	“To experience new (unknown) food”
Appearance	“From the food’s appearance?”	“The appearance of the food”
Odor	“From the food’s odour?”	“The odour of the food”
Taste	“From the food’s taste/flavour?”	“The taste/flavour of the food”
Texture	“From the food’s texture?”	“The texture of the food”
Pleased senses	“When appearance, smell and flavour perceptions are satisfied?”	“To experience a satisfaction of the sense of sight, smell, taste and touch”
Physical sensations	“From physical bodily sensations after intake?”	“To experience a positive physical sensation in the body after eating”
Mental sensations	“From mental sensations after intake?”	“To experience a positive mental feeling/sensation after eating”
Surprises	“When you are surprised about the food?”	“To experience positive surprises from the meal”
Eating w. others	“From eating in the company of others?”	“To experience eating with others”
Eating alone	“From eating when you are alone?”	“To experience eating food when I am alone”

**Table 2:** Participant characteristics.

ID	Gender	Age	Nationality *	Educational Level	Diet Type
F1	Female	25	LT	Short higher education	Flexitarian
F2	Female	22	DK	High school	Omnivore
F3	Female	24	IT	Medium higher education	Flexitarian
M1	Male	26	DK	Long higher education	Omnivore
F4	Female	49	DK	Long higher education	Vegan
M2	Male	21	PT	Medium higher education	Omnivore
F5	Female	28	DK	Long higher education	Omnivore
F6	Female	27	DK	Short higher education	Omnivore
F7	Female	28	PL	Long higher education	Flexitarian
F8	Female	28	PL	Long higher education	Omnivore
F9	Female	26	GR	Long higher education	Omnivore
F10	Female	23	DK	Vocational education	Omnivore
F11	Female	35	CA/PT	Medium higher education	Omnivore
M3	Male	23	RO	Short higher education	Omnivore
M4	Male	28	DE	Long higher education	Flexitarian
F12	Female	32	DK	High school	Carnivore
F13	Female	29	DK	Long higher education	Vegetarian
F14	Female	28	DK	Long higher education	Omnivore
M5	Male	31	DK	Long higher education	Omnivore
F15	Female	33	HU	Long higher education	Flexitarian
F16	Female	27	DK	Long higher education	Omnivore
M6	Male	26	DK	High school	Omnivore

* ‘LT’: Lithuania; ‘DK’: Denmark; ‘IT’: Italy; ‘PT’: Portugal; ‘PL’: Poland; ‘GR’: Greece; ‘CA’: Canada; ‘RO’: Romania; ‘HU’: Hungary.

**Table 3:** Overview of each participant’s individual ratings of each Food Pleasure Scale (FPS) item by the question: “How important is the following for your experience of food pleasure when eating?”. Ratings were completed by use of a 100 mm Visual Analog Scale.

FPS Items	F1	F2	F3	M1	F4	M2	F5	F6	F7	F8	F9	F10	F11	M3	M4	F12	F13	F14	M5	F15	F16	M6
Memories	33	20	100	70	74	70	69	27	60	45	87	49	100	100	50	68	80	80	18	97	24	60
Expectations	70	70	61	22	100	75	66	46	61	100	100	91	80	51	65	65	91	95	15	58	61	90
Needs	95	70	83	25	100	65	72	56	65	100	67	72	80	100	75	60	87	100	42	62	45	90
Choices	24	30	50	40	100	60	70	12	41	50	93	59	80	100	85	91	69	50	56	73	18	50
Habits	22	20	31	11	16	33	51	24	32	25	61	62	90	50	89	17	36	20	10	75	56	15
Ethical values	52	100	41	40	100	63	22	100	100	40	100	89	80	0	90	43	89	10	29	98	4	70
Prod. information	15	0	23	79	100	63	45	100	34	10	60	45	60	50	80	14	39	10	21	77	22	80
Food variation	33	100	73	100	100	52	66	77	27	80	74	84	70	75	80	81	100	30	68	86	72	70
Novelty	34	20	88	100	61	48	66	71	68	100	61	96	80	100	85	100	95	30	30	54	71	75
Familiarity	66	40	91	11	88	80	52	45	19	80	74	90	82	100	50	38	25	40	32	62	55	60
Appearance	16	70	89	13	54	85	66	74	71	100	85	79	95	100	90	26	75	50	25	76	84	70
Odor	20	90	83	100	87	83	64	69	45	100	91	80	85	75	80	82	83	90	51	89	84	70
Taste	88	100	100	100	100	96	89	100	79	100	100	93	85	100	81	100	100	100	61	96	84	90
Texture	61	100	100	60	53	95	80	75	59	100	70	78	90	100	77	100	63	90	65	66	85	80
Eating w. others	15	100	100	80	100	39	74	100	63	100	87	71	100	100	85	0	100	20	79	82	69	70
Eating alone	7	0	76	80	22	77	31	6	42	100	50	43	100	50	85	83	0	30	12	36	69	5
Physical setting	6	31	27	66	23	23	32	59	66	30	89	70	80	25	70	14	70	20	45	85	39	15
Physical sensations	5	59	86	85	83	32	71	13	37	20	70	54	70	75	90	10	56	0	26	74	31	60
Mental sensations	21	60	89	83	96	32	68	41	54	10	72	51	70	75	80	54	27	0	54	68	39	75
Surprises	3	14	93	100	86	29	71	38	39	80	95	67	80	100	90	66	40	10	27	57	73	10
Pleased senses	32	90	100	100	81	93	79	69	75	100	92	93	100	100	80	55	86	100	76	99	93	85

**Table 4:** Overview of most important Food Pleasure Scale items as rated in the questionnaire and assessed from explicit and implicit interview responses.

Most Important Food Pleasure Scale Items			
ID	Questionnaire, 4th Quartile *	Interview, Explicit Assessment	Interview, Implicit Assessment
F1	Needs, Taste, Expectations, Familiarity, Texture	Taste, Physical sensations, Expectations	Taste, Physical sensations, Eating w. others
F2	Taste, Texture, Eating w. others, Ethical values, Food variation, Pleased senses, Odor	Taste, Texture, Expectations	Eating w. others, Taste, Food variation
F3	Taste, Texture, Memories, Pleased senses, Eating w. others	Familiarity, Food variation, Novelty	Food variation, Physical setting, Taste
M1	Food variation, Novelty, Odor, Taste, Surprises, Pleased senses	Product information, Ethical values, Surprises	Taste, Pleased senses, Expectations
F4	Expectations, Needs, Choices, Ethical values, Product information, Taste, Food variation, Eating w. others, Mental sensations	Physical sensations, Mental sensations, Pleased senses	Eating w. others, Choices, Taste, Texture, Food variation, Novelty
M2	Taste, Texture, Appearance, Odor, Pleased senses	Familiarity, Expectations, Choice	Familiarity, Eating w. others, Expectations
F5	Taste, Texture, Pleased senses, Eating w. others, Needs	Eating w. others	Eating w. others, Physical setting, Taste
F6	Product information, Ethical values, Taste, Eating w. others, Food variation	Physical setting, Eating w. others, Taste, Texture	Eating w. others, Familiarity, Expectations,
F7	Ethical values, Taste, Pleased senses, Appearance, Novelty	Ethical values, Product information, Pleased senses, Taste	Eating w. others, Novelty, Familiarity, Food variation, Appearance
F8	Expectations, Needs, Novelty, Taste, Eating w. others, Appearance, Odor, Texture, Eating alone, Pleased senses	Eating w. others, Taste, Pleased senses	Physical setting, Eating w. others, Novelty, Food variation, Expectations
F9	Expectations, Taste, Surprises, Ethical values	Eating w. others, Appearance, Food variation	Eating w. others, Physical setting, Food variation
F10	Novelty, Taste, Pleased senses, Expectations, Familiarity	Eating w. others, Taste, Pleased senses	Taste, Pleased senses, Food variation, Eating w. others
F11	Memories, Eating w. others, Pleased senses, Appearance, Eating alone	Product information, Ethical values, effort	Eating w. others, Pleased senses, Novelty, Appearance
M3	Eating w. others, Familiarity, Novelty, Appearance, Taste, Texture, Surprises, Pleased senses, Memories, Needs, Choices	Eating w. others	Familiarity, Eating w. others, Appearance, Taste, Food variation
M4	Surprises, Appearance, Physical sensations, Ethical values, Habits	Physical sensations, Novelty, Familiarity	Physical sensations, Mental sensations, Texture, Taste
F12	Novelty, Taste, Texture, Choices, Eating alone	Taste, Texture, Eating alone, Novelty, Food variation	Taste, Novelty, Eating alone, Expectations
F13	Food variation, Taste, Eating w. others, Novelty, Expectations	Eating w. others	Eating w. others, Physical setting, Food variation
F14	Needs, Taste, Pleased senses, Expectations, Odor, Texture	Taste, Eating w. others, Odor, Pleased senses	Expectations, Taste, Odor, Eating w. others, Familiarity
M5	Eating w. others, Pleased senses, Food variation, Texture, Taste	Taste, Eating w. others, Pleased senses	Eating w. others, Food variation, Expectations
F15	Pleased senses, Ethical values, Memories, Taste, Odor	Eating w. others, Pleased senses, Taste	Eating w. others, Food variation, Pleased senses, Product information
F16	Pleased senses, Texture, Taste, Odor, Appearance	Appearance, Taste, Texture, Odor, Pleased senses	Eating w. others, Taste, Pleased senses, Mental wellbeing
M6	Expectations, Needs, Taste, Pleased senses, Product information, Texture	Taste, Pleased senses, Physical setting, Expectations	Eating w. others, Taste, Pleased senses, Expectations

* 4th Quartile: Items with the 25% highest importance ratings on the FPS Visual Analogue Scale.

**Table 5:** Overview of least important Food Pleasure Scale items as rated in the questionnaire and assessed from explicit interview responses.

	Least Important Food Pleasure Scale Items	
ID	Questionnaire, 1st Quartile *	Interview, Explicit Assessment
F1	Surprises, Physical sensations, Physical setting, Eating alone, Eating w. others, Product information	Product information
F2	Product information, Eating alone, Surprises, Memories, Habits, Novelty	Food variation
F3	Product information, Physical sensations, Habits, Ethical values, Choices	Product information
M1	Habits, Familiarity, Appearance, Expectations, Needs	Physical sensations, Mental sensations
F4	Habits, Eating alone, Physical setting	Familiarity, Novelty, Food variation
M2	Physical setting, Surprises, Physical sensations, Mental sensations, Habits	Physical setting
F5	Ethical values, Product information, Eating alone, Physical setting	Product information, Ethical values
F6	Eating alone, Choices, Physical sensations, Habits, Memories	Physical sensations, Mental sensations
F7	Familiarity, Food Variation, Habits, Product information, Physical sensations	Novelty, Food variation
F8	Product information, Mental sensations, Physical sensations, Habits, Physical setting	Food variation, Novelty, Familiarity
F9	Eating alone	Product information
F10	Eating alone, Product information, Memories	Product information, Familiarity, Novelty
F11	None	Expectations
M3	Ethical values, Physical settings	Physical sensations, Mental sensations, Pleased senses
M4	Memories, Familiarity	Product information
F12	Eating w. others, Physical sensation, Physical settings, Product information, Habits	Physical sensations, Eating w. others
F13	Eating alone, Familiarity, Mental sensations, Habits, Product information	None—All are important.
F14	Physical sensations, Mental sensations, Surprises, Ethical values, Product information	Physical sensations, Mental sensations, Surprise
M5	Habits, Eating alone, Expectations, Memories, Product information	Product information, Expectations
F15	Eating alone	Product information
F16	Ethical values, Choices, Product information, Memories, Physical sensations	Product information
M6	Eating alone, Surprises, Physical settings, Habits	Expectations

* 1st Quartile: Items with the 25% lowest importance ratings on the FPS Visual Analogue Scale.

## Data Availability

The data presented in this study are available on request from the corresponding author. The data are not publicly available due to the nature and detail of the qualitative data.

## Supplementary Materials

The following supporting information can be downloaded at: https://www.mdpi.com/article/10.3390/foods13030477/s1, Table S1: Overview of Food Pleasure Scale profiles of all study participants. Table S2: Overview of slides depicting the Food Pleasure Scale items. The slides were used in the qualitative interviews.

## References

[1] Dagher A. (2010). The Neurobiology of Appetite. Obesity Prevention.

[2] Köster E.P. (2009). Diversity in the Determinants of Food Choice: A Psychological Perspective. Food Qual. Prefer..

[3] Morris M.J., Beilharz J.E., Maniam J., Reichelt A.C., Westbrook R.F. (2015). Why Is Obesity Such a Problem in the 21st Century? The Intersection of Palatable Food, Cues and Reward Pathways, Stress, and Cognition. Neurosci. Biobehav. Rev..

[4] Blundell J., de Graaf C., Hulshof T., Jebb S., Livingstone B., Lluch A., Mela D., Salah S., Schuring E., van der Knaap H. (2010). Appetite Control: Methodological Aspects of the Evaluation of Foods. Obes. Rev..

[5] Dalton M., Finlayson G. (2013). Hedonics, Satiation and Satiety. Satiation, Satiety and the Control of Food Intake.

[6] Mela D.J. (2006). Eating for Pleasure or Just Wanting to Eat? Reconsidering Sensory Hedonic Responses as a Driver of Obesity. Appetite.

[7] Marty L., Miguet M., Bournez M., Nicklaus S., Chambaron S., Monnery-Patris S. (2017). Do Hedonic- versus Nutrition-Based Attitudes toward Food Predict Food Choices? A Cross-Sectional Study of 6- to 11-Year-Olds. Int. J. Behav. Nutr. Phys. Act..

[8] Czyzewska M., Graham R., Ceballos N.A. (2011). Handbook of Behavior, Food and Nutrition. Handbook of Behavior, Food and Nutrition.

[9] Andersen B.V., Chan R.C.K., Byrne D.V. (2021). A Conceptual Framework for Multi-Dimensional Measurements of Food Related Pleasure—The Food Pleasure Scale. Foods.

[10] Ruddock H.K., Hardman C.A. (2018). Guilty Pleasures: The Effect of Perceived Overeating on Food Addiction Attributions and Snack Choice. Appetite.

[11] Berridge K.C. (2009). ‘Liking’ and ‘Wanting’ Food Rewards: Brain Substrates and Roles in Eating Disorders. Physiol. Behav..

[12] Thomsen K.R., Whybrow P.C., Kringelbach M.L. (2015). Reconceptualizing Anhedonia: Novel Perspectives on Balancing the Pleasure Networks in the Human Brain. Front. Behav. Neurosci..

[13] Bédard A., Lamarche P.-O.O., Grégoire L.-M.M., Trudel-Guy C., Provencher V., Desroches S., Lemieux S. (2020). Can Eating Pleasure Be a Lever for Healthy Eating? A Systematic Scoping Review of Eating Pleasure and Its Links with Dietary Behaviors and Health. PLoS ONE.

[14] Doyon A.-A., Bédard A., Trudel-Guy C., Corneau L., Lemieux S. (2023). Adaptation and Validation of the Well-Being Related to Food Questionnaire (Well-BFQ©) for the French-Speaking General Adult Population of Québec, Canada. Nutrients.

[15] Chapman L.J., Chapman J.P., Raulin M.L. (1976). Scales for Physical and Social Anhedonia. J. Abnorm. Psychol..

[16] Chan R.C.K., Wang Y., Yan C., Zhao Q., McGrath J., Hsi X., Stone W.S. (2012). A Study of Trait Anhedonia in Non-Clinical Chinese Samples: Evidence from the Chapman Scales for Physical and Social Anhedonia. PLoS ONE.

[17] Fawcett J. (1983). Assessing Anhedonia in Psychiatric Patients. Arch. Gen. Psychiatry.

[18] Snaith R.P., Hamilton M., Morley S., Humayan A., Hargreaves D., Trigwell P. (1995). A Scale for the Assessment of Hedonic Tone the Snaith–Hamilton Pleasure Scale. Br. J. Psychiatry.

[19] Nakonezny P.A., Morris D.W., Greer T.L., Byerly M.J., Carmody T.J., Grannemann B.D., Bernstein I.H., Trivedi M.H. (2015). Evaluation of Anhedonia with the Snaith–Hamilton Pleasure Scale (SHAPS) in Adult Outpatients with Major Depressive Disorder. J. Psychiatr. Res..

[20] Franken I.H.A., Rassin E., Muris P. (2007). The Assessment of Anhedonia in Clinical and Non-Clinical Populations: Further Validation of the Snaith–Hamilton Pleasure Scale (SHAPS). J. Affect. Disord..

[21] Gard D.E., Gard M.G., Kring A.M., John O.P. (2006). Anticipatory and Consummatory Components of the Experience of Pleasure: A Scale Development Study. J. Res. Pers..

[22] Roininen K., Tuorila H., Zandstra E.H., De Graaf C., Vehkalahti K., Stubenitsky K., Mela D.J. (2001). Differences in Health and Taste Attitudes and Reported Behaviour among Finnish, Dutch and British Consumers: A Cross-National Validation of the Health and Taste Attitude Scales (HTAS). Appetite.

[23] Kowalkowska J., Lonnie M., Wadolowska L., Czarnocinska J., Jezewska-Zychowicz M., Babicz-Zielinska E. (2018). Health-and Taste-Related Attitudes Associated with Dietary Patterns in a Representative Sample of Polish Girls and Young Women: A Cross-Sectional Study (GEBaHealth Project). Nutrients.

[24] Guillemin I., Marrel A., Arnould B., Capuron L., Dupuy A., Ginon E., Layé S., Lecerf J.-M., Prost M., Rogeaux M. (2016). How French Subjects Describe Well-Being from Food and Eating Habits? Development, Item Reduction and Scoring Definition of the Well-Being Related to Food Questionnaire (Well-BFQ©). Appetite.

[25] Epel E.S., Tomiyama A.J., Mason A.E., Laraia B.A., Hartman W., Ready K., Acree M., Adam T.C., St. Jeor S., Kessler D. (2014). The Reward-Based Eating Drive Scale: A Self-Report Index of Reward-Based Eating. PLoS ONE.

[26] Vainik U., Eun Han J., Epel E.S., Janet Tomiyama A., Dagher A., Mason A.E. (2019). Rapid Assessment of Reward-Related Eating: The RED-X5. Obesity.

[27] Hyldelund N.B., Byrne D.V., Chan R.C.K., Andersen B.V. (2022). Food Pleasure across Nations: A Comparison of the Drivers between Chinese and Danish Populations. Food Qual. Prefer..

[28] Hyldelund N.B., Byrne D.V., Andersen B.V. (2022). Food Pleasure Profiles—An Exploratory Case Study of the Relation between Drivers of Food Pleasure and Lifestyle and Personality Traits in a Danish Consumer Segment. Foods.

[29] Hyldelund N.B., Frederiksen C., Byrne D.V., Andersen B.V. (2022). Is Stress Taking the Pleasure Out of Food?—A Characterization of the Food Pleasure Profiles, Appetite, and Eating Behaviors of People with Chronic Stress. Foods.

[30] Hyldelund N.B., Byrne D.V., Chan R.C.K., Andersen B.V. (2022). The Relationship between Social Anhedonia and Perceived Pleasure from Food—An Exploratory Investigation on a Consumer Segment with Depression and Anxiety. Foods.

[31] Retsinformation.dk Act on Research Ethics Review of Health Research Projects. https://www.retsinformation.dk/eli/lta/2017/1083.

[32] Zoom Video Communications Inc. Zoom Video Communications 2023. https://zoom.us/.

[33] Tong A., Sainsbury P., Craig J. (2007). Consolidated Criteria for Reporting Qualitative Research (COREQ): A 32-Item Checklist for Interviews and Focus Groups. Int. J. Qual. Health Care.

[34] Rambøll Management Consulting SurveyXact By Rambøll 2023. https://rambollxact.dk/surveyxact.

[35] Schiestl E.T., Wolfson J.A., Gearhardt A.N. (2022). The Qualitative Evaluation of the Yale Food Addiction Scale 2.0. Appetite.

[36] Kosinski M., Gajria K., Fernandes A., Cella D. (2013). Qualitative Validation of the FACIT-Fatigue Scale in Systemic Lupus Erythematosus. Lupus.

[37] Høier A.T.Z.B., Chaaban N., Andersen B.V. (2021). Possibilities for Boosting Appetite in Patients Recovering from COVID-19. Foods.

[38] Mallinson S. (2002). Listening to Respondents: A Qualitative Assessment of the Short-Form 36 Health Status Questionnaire. Soc. Sci. Med..

[39] Dikko M. (2016). Establishing Construct Validity and Reliability: Pilot Testing of a Qualitative Interview for Research in Takaful (Islamic Insurance). Qual. Rep..

[40] Kvale S. (1983). The Qualitative Research Interview. J. Phenomenol. Psychol..

[41] Braun V., Clarke V. (2006). Using Thematic Analysis in Psychology. Qual. Res. Psychol..

[42] Noble H., Heale R. (2019). Triangulation in Research, with Examples. Evid.-Based Nurs..

[43] Baumgartner H., Steenkamp J.-B.E.M. (2001). Response Styles in Marketing Research: A Cross-National Investigation. J. Mark. Res..

[44] Fadnes L., Taube A. (2012). How to Identify Information Bias Due to Self-Reporting in Epidemiological Research. Internet J. Epidemiol..

[45] Beard C., Björgvinsson T. (2014). Beyond Generalized Anxiety Disorder: Psychometric Properties of the GAD-7 in a Heterogeneous Psychiatric Sample. J. Anxiety Disord..

[46] Thorsteinson T.J. (2018). A Meta-analysis of Interview Length on Reliability and Validity. J. Occup. Organ. Psychol..

[47] Barkus E. (2021). The Effects of Anhedonia in Social Context. Curr. Behav. Neurosci. Rep..

[48] Klaaren K.J., Hodges S.D., Wilson T.D. (1994). The Role of Affective Expectations in Subjective Experience and Decision-Making. Soc. Cogn..

[49] Herman C.P., Polivy J. (2005). Normative Influences on Food Intake. Physiol. Behav..

[50] Meiselman H.L., Johnson J.L., Reeve W., Crouch J.E. (2000). Demonstrations of the Influence of the Eating Environment on Food Acceptance. Appetite.

[51] Spencer J. (2021). The Sustainable Development Goals. Design for Global Challenges and Goals.

[52] Cardello A.V., Schutz H., Snow C., Lesher L. (2000). Predictors of Food Acceptance, Consumption and Satisfaction in Specific Eating Situations. Food Qual. Prefer..

[53] Lawless H.T., Heymann H. (2010). Sensory Evaluation of Food.

[54] Andersen B.V., Brockhoff P.B., Hyldig G. (2019). The Importance of Liking of Appearance, -Odour, -Taste and -Texture in the Evaluation of Overall Liking. A Comparison with the Evaluation of Sensory Satisfaction. Food Qual. Prefer..

[55] DeVellis R.F., Salmon H. (2017). Scale Development—Theory and Applications.

[56] Dutil É., Bottari C., Auger C. (2017). Test-Retest Reliability of a Measure of Independence in Everyday Activities: The ADL Profile. Occup. Ther. Int..

