# Supplementary material for: Measuring Pleasure from Food—Validation of the Food Pleasure Scale by Multiple Techniques and Mixed Methods
Source: Foods. 2024 Feb 2;13(3):477. doi: 10.3390/foods13030477 (PMC10855913; doi:10.3390/foods13030477)
Supplement: Supplementary file 1 [file foods-13-00477-s001.zip › Table S1.pdf]

Table S1. Overview of Food Pleasure Scale profiles of all study participants

<table>
<tr><th>ID</th><th>Food Pleasure Scale profile</th><th>Most important Food Pleasure Scale items, Questionnaire, 4th quartile*</th></tr>
<tr><td>F1</td><td>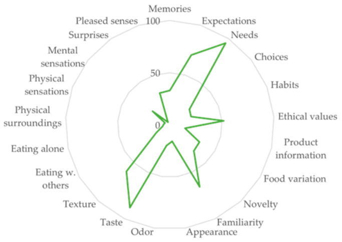</td><td>Needs, Taste, Expectations, Familiarity, Texture</td></tr>
<tr><td>F2</td><td>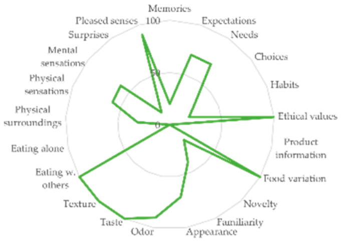</td><td>Taste, Texture, Eating w. others, Ethical values, Food variation, Pleased senses, Odor</td></tr>
<tr><td>F3</td><td>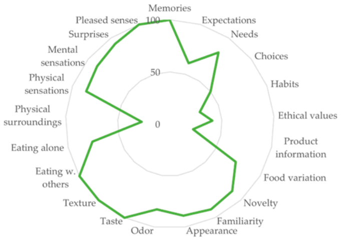</td><td>Taste, Texture, Memories, Pleased senses, Eating w. others</td></tr>
<tr><td>M1</td><td>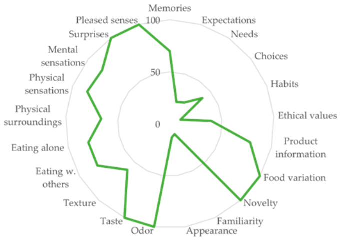</td><td>Food variation, Novelty, Odor, Taste, Surprises, Pleased senses</td></tr>
<tr><td>F4</td><td>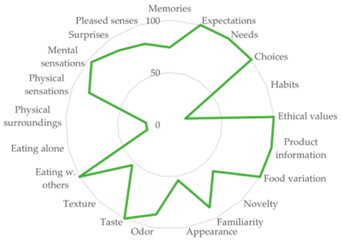</td><td>Expectations, Needs, Choices, Ethical values, Product information, Taste, Food variation, Eating w. others, Mental sensations</td></tr>
</table>

**M2**	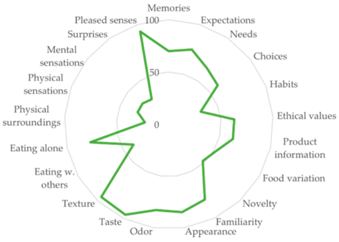	Taste, Texture, Appearance, Odor, Pleased senses
**F5**	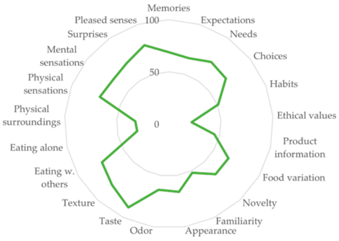	Taste, Texture, Pleased senses, Eating w. others, Needs
**F6**	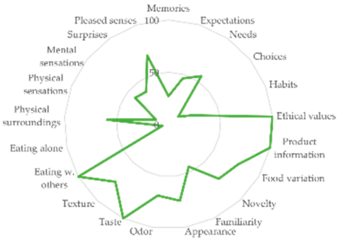	Product information, Ethical values, Taste, Eating w. others, Food variation
**F7**	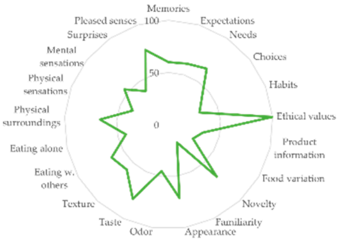	Ethical values, Taste, Pleased senses, Appearance, Novelty
**F8**	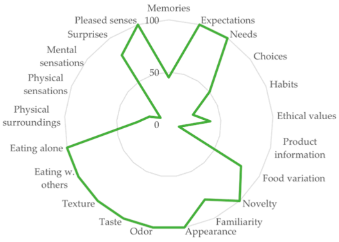	Expectations, Needs, Novelty, Taste, Eating w. others, Appearance, Odor, Texture, Eating alone, Pleased senses
**F9**	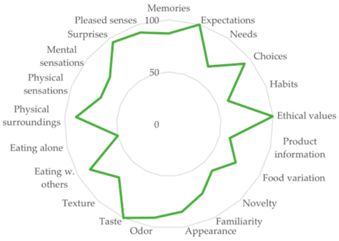	Expectations, Taste, Surprises, Ethical values

**F10**	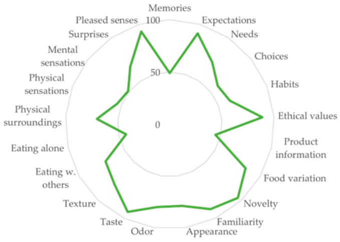	Novelty, Taste, Pleased senses, Expectations, Familiarity
**F11**	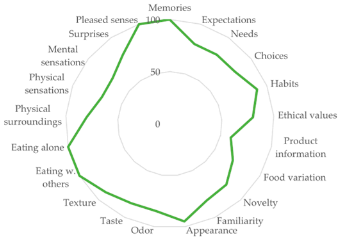	Memories, Eating w. others, Pleased senses, Appearance, Eating alone
**M3**	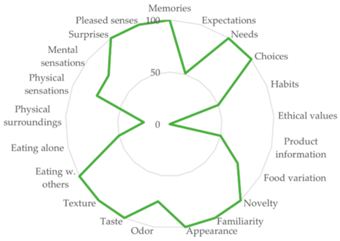	Eating w. others, Familiarity, Novelty, Appearance, Taste, Texture, Surprises, Pleased senses, Memories, Needs, Choices
**M4**	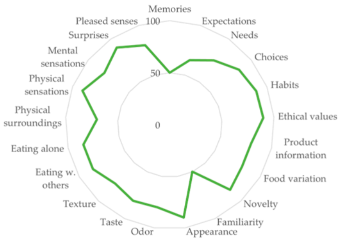	Surprises, Appearance, Physical sensations, Ethical values, Habits
**F12**	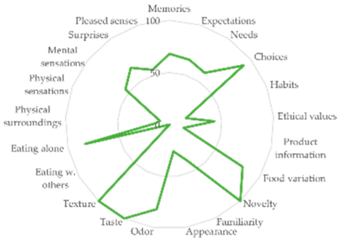	Novelty, Taste, Texture, Choices, Eating alone
**F13**	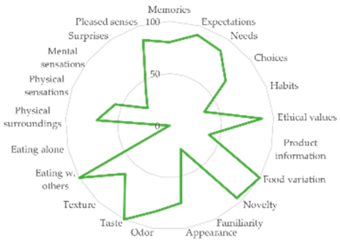	Food variation, Taste, Eating w. others, Novelty, Expectations

<table>
<tr><td>F14</td><td>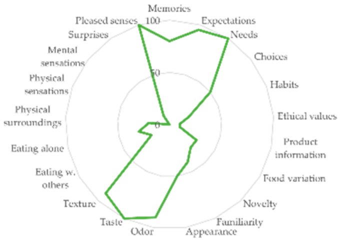
</td><td>Needs, Taste, Pleased senses, Expectations, Odor, Texture</td></tr>
<tr><td>M5</td><td>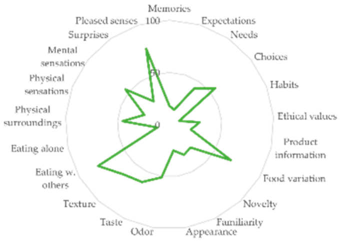
</td><td>Eating w. others, Pleased senses, Food variation, Texture, Taste</td></tr>
<tr><td>F15</td><td>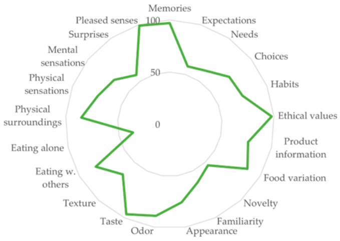
</td><td>Pleased senses, Ethical values, Memories, Taste, Odor</td></tr>
<tr><td>F16</td><td>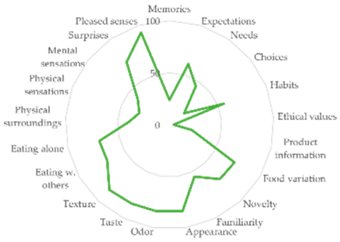
</td><td>Pleased senses, Texture, Taste, Odor, Appearance</td></tr>
<tr><td>M6</td><td>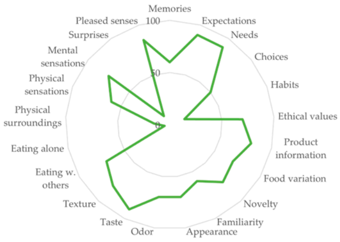
</td><td>Expectations, Needs, Taste, Pleased senses, Product information, Texture</td></tr>
</table>

*4th Quartile: Items with the 25% highest importance ratings on the FPS Visual Analogue Scale.
