# Supplementary material for: Measuring Pleasure from Food—Validation of the Food Pleasure Scale by Multiple Techniques and Mixed Methods
Source: Foods. 2024 Feb 2;13(3):477. doi: 10.3390/foods13030477 (PMC10855913; doi:10.3390/foods13030477)
Supplement: Supplementary file 1 [file foods-13-00477-s001.zip › Table S2.pdf]

Table S2. Overview of slides depicting the Food Pleasure Scale items. The slides were used in the qualitative interviews.

Topic	Slide shown during interviews
Sensory properties	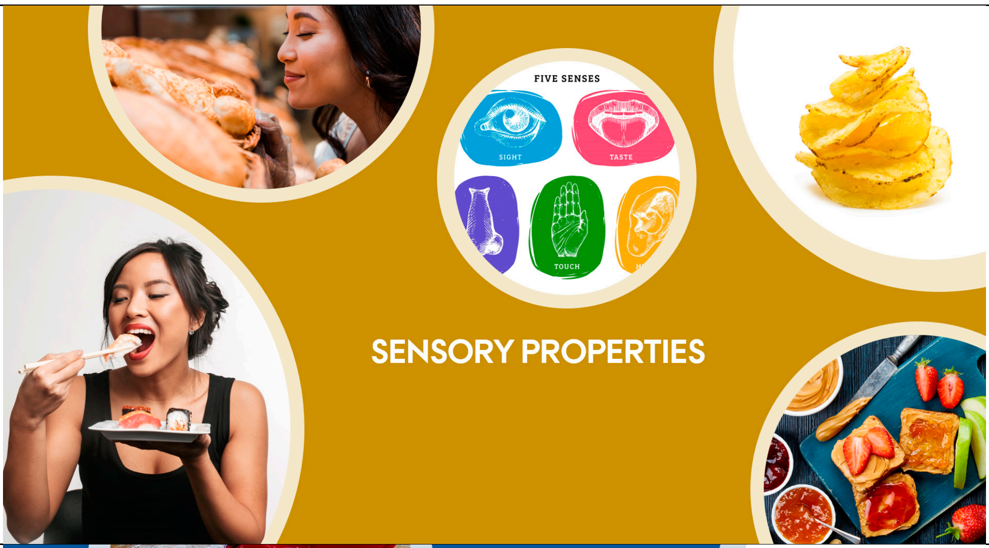
Expectations and desires	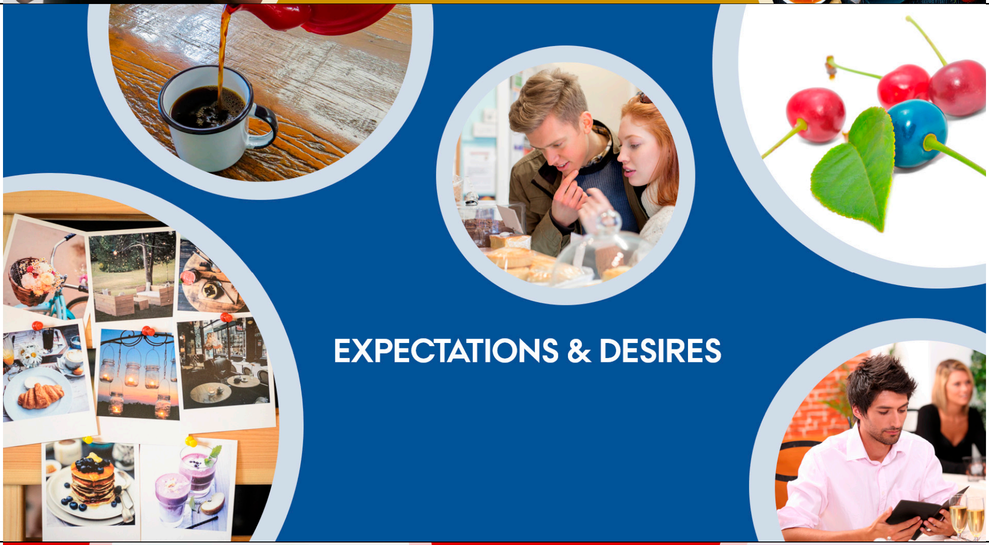
Collative properties	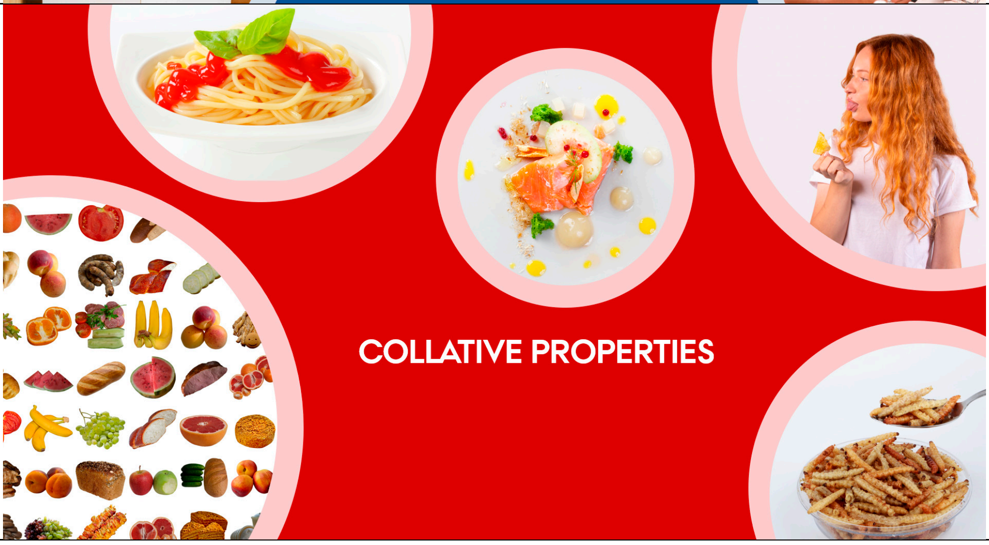

Eating context		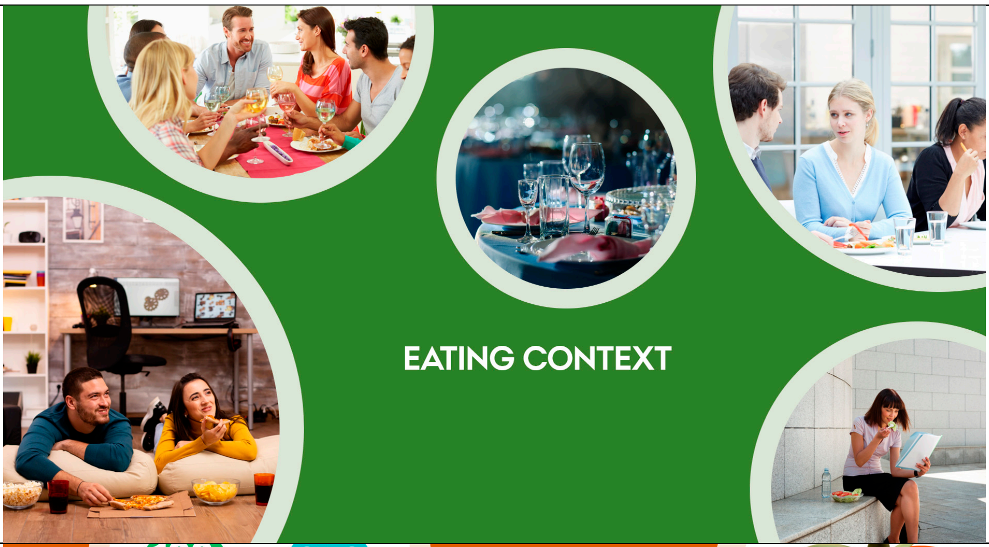
Product information		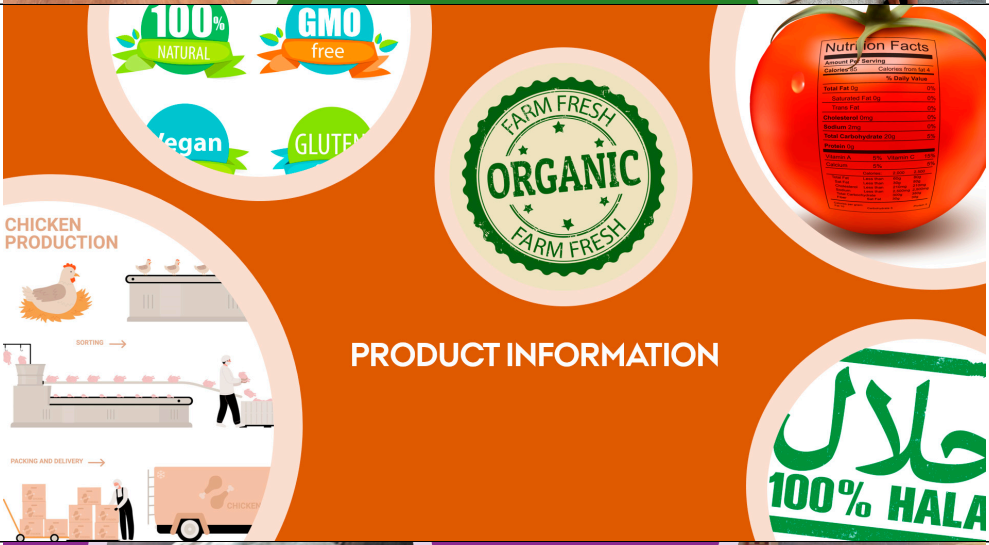
Post-ingestive sensations		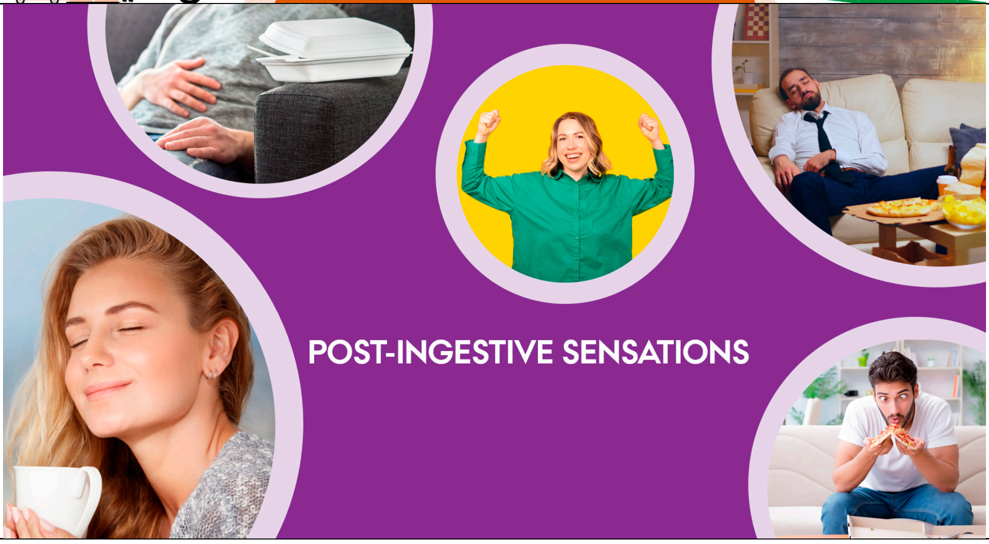

Figures adapted with permission from Copyright 2023 Colourbox.
